# On the Definition of Hypnosis

**Published:** 1896-06

**Authors:** Max Hirsch

**Affiliations:** Berlin


### ON THE DEFINITION OF HYPNOSIS.*

By DR. MAX HIRSCH,

BERLIN.

To unite an idea with a word is as old as our language. New words are only formed whenever it becomes necessary to agree on an exact term for an unknown idea. This should be especially so in science. If in this connection we consider the word "hypnosis," we should think that a state is meant showing a relation to normal sleep (ὑπνος). Against that, Bernheim, one of the founders of this healing method, asserts *Hypnosis exists without sleep;* with that he will express, that a number of so-called hypnotic phenomena can be produced on some persons when awake.

In my text-book† I proposed, in order to prevent a confusion of ideas which from this has to result with certainty, to call that condition in which an individuum has taken a suggestion when waking and conscious captivation. To what would it lead if we should consider every man hypnotized who has taken a suggestion? Yet such a nonsense seems to become usage in our language. Frequently we read in journals, novels, etc., this or that man was completely hypnotized instead of "fascinated," which was formerly used.

*Translated from *Deutsche Medicinal Zeitung*, 91, 1895, by Dr. Otto Lerch, New Orleans.

†*Suggestion und Hypnose*, Leipzig, 1893.—Abel.

What is meant then by hypnosis? What are the characteristic signs of this condition? If these questions could be answered shortly and precisely a great service would be rendered to the suggestion therapy, and the following discussion would be superfluous. Such an answer, however, is impossible to give. On reading the various definitions of the authors one cannot help but feel that they mostly try with complicated phrases to get around a clear and simple definition, and at the same time one becomes conscious of knowing less of the hypnotic state than before. The reason for the boundless confusion and unclearness is, in my opinion, solely due to the fact that it has been tried to call a number of various conditions, which have nothing or little to do with each other, by one collective name. After it was once discovered that in the hypnotic state at one time a certain symptom and then again another symptom became apparent, one was forced to divide the state in various degrees. A condition in which the hypnotized took but a few suggestions was considered to be a light degree, and that in which all symptoms of hypnosis were present was called a deep hypnosis. Various marks of distinction were given to be characteristic for each degree. Krafft-Ebing notices two, Ford and Charcot three, Liébault five, Bernheim nine degrees of hypnosis. One author sees the mark of distinction in the power to open or shut the eyes; another in the present or absent amnesia, etc. Mostly it is spoken of as a light or a deep sleep, and it is far too little considered by the authors that but in a few cases sleep comes in question at all.

What I think of the hypnotic condition after an experience gained from thousands of practical experiments, I wish here to state in short: In my opinion *hypnosis as an independent state does not exist*, and it would be desirable to strike this word entirely, which has caused so much trouble, and has made the suggestion therapy so many enemies. However, this is hardly possible at present, and it is therefore necessary to see what the state of hypnosis really is.

First, there are a number of persons who, upon the sleep suggestion thought to produce hypnosis, do not sleep, and tell this to the physicians. The expert will then either try further experiments to have the suggestions accepted, or he will be satisfied with a state of calmness of mind (*Gemucth sberuhigung*) with eyes closed, in which condition by far the most therapeutic suggestions can be brought to act. It is especially easy to produce with suggestion euphoria, at least a melioration of humor as well as to free from pain, and that frequently in cases in which a corresponding suggestion without a previous calming of mind and without eye-shutting had been in fruit unsuccessful. With this, quite a large number of cases are settled in which the authors speak of hypnosis. Many use exclusively this condition for therapeutic purposes. Professor Hirt, for instance, declares that for therapeutic use this state is sufficient and that his patients are never brought into any other. I too have found this condition in many cases entirely sufficient in suggestive treatment; was, however, always conscious that hereby a hypnotic therapy could not be spoken of. As a proper name for this special state of hypnosis, I would suggest a term which occasionally has been used in literature synonymously with hypnosis: "Passive rest of Mind." Of nothing else can be the question in this condition. The theory how in such cases the heightened suggestibility is to be explained cannot be treated of in this paper. That it is present and that it explains the acceptation of many suggestions, and therefore, the useful application of this condition in suggestion therapy cannot be doubted. According to my calculations, which however do not pretend to be exact, a sleep suggestion will produce only this state in about twenty-five per cent. of men.

Contrary to this condition in which the acceptation of the sleep suggestion is not produced, is that state in which that suggestion is accepted. The persons come in consequence of the sleep suggestion to sleep illusion. This state can be produced in the majority of men, about sixty per cent. The attention becomes fixed on the idea of sleep upon which it is concentrated during that state.

Through this averting of attention the control of brain activity is diminished so that suggestions are readily accepted. Mobility, sensibility, and common sensation are especially influenced almost by all persons who are in the state of sleep illusion. Rarely, and then only in a small degree, the sensorial sphere. How different, however, is this state from the foregoing (passive rest of mind), and yet it is called, like that, hypnosis. In consequence one has to speak in such a case of a second degree of hypnosis, as in this condition more suggestions are accepted, and most of all the suggestion of sleep. This does not mean, however, that the persons actually sleep. The acceptation of the suggestion of sleep takes place in a waking condition. The persons are and remain awake and keep their normal consciousness.

What, then, is the state in which one can put a person in all possible positions, influence their memory, attack the consciousness of their very self (seh-Bewuntsein), and after which a spontaneous amnesia exists for everything experienced in that condition. The authors assure us *"that is the deepest degree of hypnosis; that is hypnosis."* Nay, that is not hypnosis at all, that is sleep, the same sleep these persons sleep at night. Of course it is a peculiar sleep. One can converse with these persons during their night sleep as well, and they accept all possible suggestions during that sleep. This sleep has been produced with suggestion, just so as these persons, where and when they wish, can produce it, whenever they have the mere intention to sleep. It is the same, however, whether I give the suggestion of intention to sleep to a person, or whether that person takes that intention without my suggestion. After all, each suggestion has to be followed by auto-suggestion before it can act. I have spoken about this sleep, which I have called "somnambulic sleep" (somnambuler schlaf), after having had occasion to make this remarkable observation on a number of persons, in another paper, to which I hereby refer.*

What has this condition, which we find in about 10 per cent. of men, in common with that previously discussed? This state is something peculiar; it represents a changed position of consciousness (veraenderte Bewusstscinslage), opposite the waking condition. The only thing that state 1 and state 2 have in common is their production through sleep suggestion, whereas all three states participate equally only in the heightening of suggestibility; though

*Neber Schlaf, Hypnox und Somnambulismus, *Deutsche Med. Wachenschrift*, 36, 95.

one might perhaps add the external similarity, rest position with shut eyes, but even this mark can be changed through suggestion. It remains, then, only to remark that a relatively small percentage of men, about five per cent., cannot be influenced at all through sleep or calming suggestion. In that case it is said that these persons cannot be hypnotized. Many authors have not considered the first state as hypnosis, which seems reasonable enough. These have given the percentage of persons not to be hypnotized to be 20 to 30 per cent., whereas others, in opposition to this, have maintained that only five per cent. cannot be hypnotized, due to the fact that they have regarded the first state (passive rest of mind) as a degree of hypnosis, and by no means due to their greater ability to produce hypnosis.

If we consider all that is said above, we find that the first degree is nothing but an artificial calming of mind; the second degree, the belief to sleep, acquired in the waking condition; the third degree, however, a peculiar sleep.

What, then, is hypnosis? It seems to me that it was the third degree that has caused the study of the remarkable phenomena, as "the magnetiseurs" could produce their interesting experiments only on persons in this state, and these persons were called media. Not knowing that this condition was a mere species of sleep, it was called with a new name, hypnosis. Later, this term was extended to cover all the conditions above discussed, which were thought to be lighter degrees of hypnosis. We have seen that this view is untenable, further shown by the fact that the first two degrees can never be converted into the third one.

Finally, the question arises, shall we, knowing how badly the new term has been selected, and for how many different conditions it is used, keep it, or dispense with it? As mentioned before, I would like to advocate to strike the word; yet I believe this will be, considering the present literature and the habit, almost impossible. I would suggest, however, to call the third degree, entirely different in principle, "*somnambul-hypnosis*." When we, then, bear in mind that by hypnosis we have nothing but a heightened suggestibility, by mostly waking consciousness, the confusion will be less than it has been heretofore, and the nimbus attached to the word hypnotism will be greatly lessened if not destroyed.

